# The Object of Our Profession

**Published:** 1872-11

**Authors:** C. P. Baird


### The Object of Our Profession.

BY DR. C. P. BAIRD.

*Read before the Tennessee Dental Association.*

We are to understand that there is an object for every one who wishes to honor his profession, or have his profession honor him. How often is it the case that young men start out in life without any fixed purpose in view, to be driven to and fro like a ship cut loose from its moorings, to be lashed by the tempest, and born away upon an angry ocean without compass or chart, to tell whither bound. For we must be admonished at all times by those who have passed before us, and have left landmarks by the way to guide our erring footsteps in the way that tends upward and to honor.

So he who starts out in professional life has spread out before him the map of many generations, so well marked that he cannot, with reason to admonish, depart from the way; and yet how frequent, we hear the expression, that we are not responsible for the acts of others. This is not true, but the reverse, for we are all moulded to a greater or less degree by the circumstances that surround us. And hence the responsibility that rests upon each of us is of great magnitude, and should be felt in all the transactions of life. It is also a fearful thought that in the sequel of our days we will have to render an impartial account.

The diversified tastes and dispositions of men are great indeed, for in all the circles of life we see this demonstrated most clearly in every day's association; yet we are only admonished by this that we in all onr efforts at perfection too plainly show the want of it.

Now let us turn our eyes upon the surrounding world, if we wish to see a true type of ourselves. How often is it the case that in passing judgment upon others we only bring to light our own condemnation. This does not only hold good in individuals, but professionally.

We are apt to think that we are far in advance in many respects of those who have preceeded us. Whilst this may be true to some degree, still we find the mass of superstition continues to grow, and if we choose to remain in idleness we will soon find its gloomy festooned drapery hanging in thick folds about us also. And yet an open door still invites us to enter; and the hand of science points to fields of a rich harvest, where the voice of the joyous reaper has not yet been heard.

Some seem to stop as it were in their march, whilst the meridian sun in full glory above them is calling aloud for science and art to put forth their hands and receive that which is more than golden treasure.

The incentive is just as strong now as it has ever been for those who live for the welfare of our once happy race, as it came from the hand of the Great Architect. But how changed and distorted is every lineament of feature that once was the express image of godlike perfection, but is now transformed when unrestrained into the likeness of the old arch-fiend that Milton describes, on his way to a world of misery and woe, whose inheritance has become infected with the malady of sin, and left to mourn our better days that took their flight from Eden's happy bowers. Finally, my brethren, let us

"So live that when our summons comes to join
The innumerable caravan, that moves
To the pale realms of shade, where each shall take
His chamber in the silent halls of death,
We go, not like the quarry slave at night,
Scourged to his dungeon, but, sustained and soothed
By an unfaltering trust, approach our graves
Like one who wraps the drapery of his couch
About him, and lies down to pleasant dreams.

